# Optometry clinical training and practice at the public health facilities in South Africa: a documentation review

**DOI:** 10.4314/ahs.v26i2.20

**Published:** 2026-06

**Authors:** Raserogole F Segooa, Vanessa R Moodley

**Affiliations:** 1 Discipline of Optometry, School of Health Sciences, University of KwaZulu-Natal, Durban, South Africa; 2 Department of Optometry, Department of Health, Mokopane Hospital, Limpopo, South Africa

**Keywords:** Optometry student, clinical training, clinical supervision

## Abstract

**Background:**

Three bodies involved in the training of health professionals in South Africa, the Health Professions Council of South Africa, Council for Higher Education and the Department of Health, are expected to align and meet the quality education, training and practice standards of health professions, including clinical training and practice of optometry students at public health facilities.

**Objectives:**

To review documents related to optometric student clinical training standards in South Africa, to determine their alignment and ascertain whether they serve their intended purpose.

**Methods:**

A qualitative documentation review study using content analysis to collect, explore and compare data from purposively sampled documents, apraised using the international centre for allied health evidence instrument.

**Results:**

Two themes emerged from the review, namely; the required optometry knowledge, skills and conduct and the required optometry processes and procedures. However, the frameworks of the reviewed documents vary vastly in terms of quality, transparency and core content.

**Conclusion:**

There is general alignment of the defined clinical training and supervision standards amongst universities offering optometric education and the regulatory authorities within the public health facilities in South Africa. There is still a need to standardise the documents guiding optometry student clinical training and practice, with clear, consistent, referenced, accurate and evidence-based recommendations.

## Introduction

Clinical training is a central component in health professions, education and an integral bridge between educational and professional environments^1^. In addition to universities offering the health professions qualifications, various bodies are tasked with ensuring that health programmes are of a high quality. In South Africa (SA), they are the Health Professions Council of South Africa (HPCSA), Council on Higher Education (CHE) and the Department of Health (DOH). The HPCSA regulates education, training, registration and practicing of health professions registered under the Health Professions Act No. 56 of 1974, and provide for matters incidental thereto^2^. CHE promotes quality assurance in higher education, audits the quality assurance mechanisms of higher education institutions and accredits higher educational programmes through the Department of Higher Education and Training (DHET)^3^. The DOH provides a framework for a structured and uniform Health system^4^.

In recent years, there has been an increase in emphasis on the setting and measurement of quality standards across all sectors, including those involved in health education and service. As there are different stakeholders involved in the setting, implementation, monitoring and assurance of standards and quality in clinical training, one will expect that there be collaboration and alignment of policies, procedures and guidelines. It is important that evidence-based guidelines developed adhere to particular standards, to the extent possible, to ensure robust and comprehensive support for the recommended actions^5^. In Canada, the Council on Education for Public Health 5 requires that health education schools function as a collaboration of disciplines, and provide a learning environment that supports interdisciplinary communication. Furthermore, to foster this collaboration, training in inter-professional education is recommended to be included in the Council's accreditation standards for optometry programs^5^.

Clinical training for optometry students in South Africa occurs both within the institution clinics and at DOH facilities. It is unknown whether there is alignment of student training protocols, clinical competency outcomes and practice standards at DOH facilities, against defined clinical training competency standards of universities and the HPCSA's professional board for optometry and dispensing opticians (PBODO).

### Research problem

The four universities offering optometry programmes in SA are University of Free State (UFS), University of Johannesburg (UJ), University of Limpopo (UL) and University of KwaZulu-Natal (UKZN). To comply with the university clinical programme and the HPCSA minimum competency standards requirements, optometry students have been undertaking clinical training at public sector clinics and/or hospitals in recent years^6^. There is currently no scholarly evidence of alignment of the defined optometry clinical competency criteria used by SA universities and the DOH standards for clinical learning. It is also unknown whether there is collaboration amongst the relevant regulatory bodies (HPCSA and CHE), the DOH and training institutions, in relation to clinical quality and minimum standards or, whether any standardized system is used at public health facilities for student clinical training.

Anecdotal reports suggest that DOH-based student supervisors remain frustrated with having to undertake supervision, not knowing exactly what is expected of them and which skills are needed to undertake clinical supervision of optometry students. They often articulate these frustrations by empathically declaring that: “they are clinicians and not educators”. Further, they report attempting to provide supervision in accordance with what they may feel is “the best of their ability”, with uncertainty as to whether their efforts meet the required academic standards. The aim of this study was to review the documents related to standards for optometry clinical training of the HPCSA, CHE and DOH, and compare them with the defined clinical training standards, protocols and guidelines of the universities offering optometry programmes in SA.

## Ethical consideration

Ethical clearance was obtained from the University of KwaZulu-Natal, Humanities and Social Sciences Research Committee; reference number HSSREC/00002829/21. The study complied with the tenets of the Helsinki declaration. It viewed the participants as autonomous agents with the right to voluntarily accept or decline to participate and cease at any stage^7^.

## Methods

A literature review can serve as a basis for development of knowledge and quality guidelines^8^. A qualitative, comparative design was followed to review the commonalities and differences in the policies, procedures, guidelines, standards and protocols for learning and assessments used in optometric clinical education in South Africa. Sampling in qualitative research is about appropriateness, purpose and access to rich information^9^. Purposive sampling was conducted in two stages to source appropriate optometry documents for this study. First, the CHE, HPCSA and National DOH websites were visited to search for appropriate optometry documents in optometry student clinical training and practice at DOH facilities; where documents are not available online, the responsible administrative personnel were contacted to request access to the required documents. In stage two, snowball sampling was conducted through the head of optometry departments of the four universities offering optometry programs in SA, who referred the researchers to the relevant staff involved in co-ordinating student clinical training, who provided the researchers with existing documents (guidelines, forms, record cards, patient log-sheets etc.) used for optometry student clinical training at DOH facilities.

Research on content analysis of a variety of clinical guidelines reveal that guidelines in general are regularly developed and proven beneficial, however, they are widely underused and thus not achieving the benefits that they are intended for^10^. Data on student clinical training was collated and compared to determine whether each university's optometry student clinical training guidelines and defined standards for use at the public health facilities align with each other and with the CHE, DOH and PBODO policies and/or guidelines. Documents not directly addressing or used in optometry student clinical training at DOH facilities were excluded from the study.

### Document Analysis

A variety of documents were sourced, but for the purpose of this study, a general term ‘document’, will be used to describe each, irrespective of whether it is referring to a policy, protocol, clinical guideline, competency standard, standard operating procedure, manual or assessment form. Documents sources were and analysed in two distinct phases: document appraisal and content analysis. The international centre for allied health evidence (iCAHE) quality checklist was used for appraisal because the instrument provides an efficient quality rating process per guideline, and is a clinically acceptable alternate to the other time consuming instruments^11^, such as the appraisal of guidelines research and evaluation of health systems (AGREE-HS). Content analysis was conducted according to a guide by Erlingsson and Brysiewicz, which systematically transforms large amounts of extracted texts from the sourced documents, into highly organised and concise summary of key results^12^. These phases considered elements of the READ approach, which systematically collect documents and gain information from them in the context of health policy studies at any level^13^.

### Document appraisal

There is no standard approach to assess the quality of guidelines that addresses the needs of researchers, methodologists, educators, clinicians, policy-makers and managers^11^. For this reason, the researchers chose the iCAHE instrument (Source 1). The instrument consists of 14 questions. A score of “0” is applied for a “no” answer, “1” for a “yes” answer. The total of yes scores divided by the maximum score of 14 gives the document score, expressed as a percentage.

### Content analysis

Krippendorff (2004) and Downe-Wambolt (1992), quoted in Bengtsson (2016), define content analysis as a research technique that provides a systematic and objective means to make replicable and valid inferences from texts (or other meaningful data), to the context of their use, and describe a specific phenomenon^14^. On the other hand, Sandstrom describes content analysis as flexible in terms of the research design, using either a deductive and/or inductive approach as determined by research purpose^15^. Its definitions and flexibility led the researchers in choosing this method to review data and determine the alignment of sourced documents amongst each other, and against the regulators' requirements.

The primary researcher guided and supported by the supervisor gathered information from sourced documents, which were summarised according to their meaning, for ease of reference, thus enabling extraction of similar data (Table 1). Data was initially divided into meaningful units, which were further condensed and labelled through formulation of codes, and their patterns compared, organised and categorised into themes (Table 3). It is recommended that experts be involved in content analysis as this process required several iterations in understanding the extracted data to establish themes and subthemes^7^. It requires someone who will be able to produce, develop and understand the empirical knowledge objectively^16^. It is for this reason that the researchers repeatedly read, reviewed and refined themes in comparison of all data types.

**Table:** 

Guideline:	
Guideline producer:	
Link:	
Is the guideline readily available in full text?	(1)
Does the guideline provide a complete reference list?	(1)
Does the guideline provide a summary of its recommendations?	(1)
**Dates**	
Is there a date of completion available?	(1)
Does the guideline provide an anticipated review date	(1)
Does the guideline provide dates for when literature was included?	(1)
**Underlying Evidence** Does the guideline provide an outline of the strategy they used to find underlying evidence?	(1)
Does the guideline use a hierarchy to rank the quality of the underlying evidence?	(1)
Does the guideline appraise the quality of the evidence which underpins its recommendations?	(1)
Does the guideline link the hierarchy and quality of underlying evidence to each recommendation?	(1)
**Guideline developers** Are the developers of the guideline clearly stated?	(1)
Does the qualifications and expertise of the guideline developer(s) link with the purpose of the guideline and its end users?	(1)
**Guideline purpose and users** Are the purpose and target users of the guideline stated? **Ease of use**	(1)
Is the guideline readable and easy to navigate?	(1) **TOTAL/14**
**Score**	

Source 1: International Centre for Allied Health Evidence (iCAHE) City East Campus, North Tce, Adelaide University of South Australia

**Table 1:** Summary of 12 documents reviewed

Source/Authors	Audience/Target	(Document number) & Title	Purpose &/or objectives of the document	Results (outcomes, interventions &/or recommendations)
PBODO DOCUMENTS	Universities	(1) Optometry exit level outcomes core competencies	Not indicated	The document outlines the core fields upon which students must demonstrate competence and be evaluated. The required core fields are: - cross field competencies (communication skills and professional conduct) - clinical competencies (visual function, optical appliances, ocular examination, ocular abnormalities, paediatric vision, contact lenses and binocular vision)
		(2) Guideline for evaluation and accreditation of clinical training facilities	To ensure that graduates of accredited programmes are educated and trained in a core set of knowledge, attributes and skills required for competent, ethical, safe and effective independent professional practice. To foster continuous enhancement of optometric education and training in South Africa in terms of clinical optometry practice, universities' community outreach service and student exposure to workplace learning.	The document directs the evaluators of the clinical training facilities to verify the accuracy of completed self-evaluation forms by the institutions to: - ascertain the range of equipment for associated examinations that will enable students to gain clinical practice and develop clinical competence in relation to the approved curriculum; - determine whether there is available general clinical equipment, special clinics equipment, and dispensing equipment. - determine the level of clinical supervision and accompaniment of the students by all members of staff, and that there are suitably qualified supervisors with sufficient relevant experience and mentoring/coaching competence. - ascertain the level to which the clinical training department promotes an atmosphere that is conducive to student learning, by implementing effective teaching and learning methods, availability of learning materials and suitable learning opportunities to facilitate the purpose and outcomes of the programme. - verify that a system for clinical assessment is in place to enable students in managing the clinic and patients, which includes availability of basic equipment, clinical record cards to document patient demography, case history, clinical procedures, vision and ocular health assessment findings, diagnosis and management plans. - verify that the communication channels between the clinical training department and the training institution promote and assure the quality of student clinical training, by ensuring availability of programme coordinator and clinical training policies.
		(3) Guidelines for recognition of clinical hours and cases for optometry students	To enable a monitoring and quality assurance process in the recognition of clinical hours and patient numbers acquired by optometry students by higher education institutions. To ensure consistency in the application of the established criteria during the clinical training of optometry students.	Clinical hours are recognised when students perform or get exposed to one or more of the following criteria: - clinical competencies (supervised patient examination, investigative techniques, therapeutic procedures, patient health screening, optical device management, vision therapy) - cross fields competencies (patient education, patient skills training, case observation and clinic/practice administration)
	Universities and Practitioners	(4) The Basic Eye Exam	Not indicated	The document outlines the minimum clinical optometric procedures and equipment checklist required to conduct a basic eye examination, as well as the minimum optical dispensing requirements. It includes some of the critical cross-fields and clinical outcome competencies.
	Foreign qualified optometrists who wish to register to practice in SA SA optometrists who were off-register for more than 5 years	(5) Guidelines for the board examination	To assist an applicant who wishes to register as an optometrist with the PBODO of the HPCSA in preparation for the National Board examination. To outline the core competencies that the candidate will be evaluated on during the national board examination. To guide the examiners and moderators in preparation for the national board examination and during assessment.	The documents outline the evaluation process of determining the candidate's knowledge and proficiency (ability to demonstrate and perform the clinical procedures) in core competencies: - cross fields competencies (communication skills & professional conduct) - clinical competencies (visual function, visual aids &/or other management strategies, ocular examination, ocular abnormalities, contact lenses and binocular vision)
	SA optometrists who have not been practising within the profession for more than five (5) years	(6) Guidelines for supervised practice	To provide supervised practice guidelines to practitioners registered under supervision (“supervisees”) and registered practitioners who will be supervising the supervisees.	The document outlines the expected outcomes to be met after the practitioner has undergone supervised practice, and they include: - cross fields competencies (clinical practice, patient management, general clinical skills, practice management and ethics) - clinical competencies (ocular diagnostic procedures, contact lenses, paediatric optometry, binocular vision, low vision, ocular disease diagnosis and management, ocular therapeutics and optical dispensing) The document also provides the responsibilities of both the supervisor and the supervisee, as well as what to record and report on (i.e. log-sheet and reporting template).
UNIVERSITY DOCUMENTS	Clinical supervisors	(7) General clinic evaluation form [University 1]	Not indicated	The document outlines the assessment requirements for general optometry clinic, namely: - the average duration a student should take to examine the patient - supervisor/assessor/examiner information and instructions on how to assess a student, including mark allocations - a breakdown of possible procedures and tests a student is expected to perform
	Supervisors and students	(8) Patient report/record card [University 2]	Not stated	The document outlines predetermined procedures and techniques a student must perform on patients and record findings. It has a section for the supervisor to sign-off, confirming observation of the student's clinical and cross-field competencies. The record cards vary for different clinics, namely: - General optometry - Contact lens - Binocular vision - Low vision - Paediatric optometry clinic
	Decentralised Clinical Training (DCT) Optometrists	(9) Decentralised clinical training platform: General clinic guidelines [University 3]	Not stated	The document outlines: - expected student responsibilities and conduct while on site - supervisor information on what to expect from students - supervisor instruction on how to train and supervise students - expected minimum procedures and techniques a student must conduct when examining general optometry clinic patients, as prescribed by the HPCSA (basic examination guidelines) - expected minimum procedures and techniques a student must conduct when conducting specialised optometry clinics, as prescribed by the HPCSA, that is, binocular vision, low vision, paediatric optometry, disease management and contact lens - assessment requirements including log-sheet and marks allocation
	DCT Optometrists	(10) Student evaluation form [University 3]	Not stated	The document outlines clinical and cross fields competencies to rate the performance of a student by the end of a block, based on the following criteria: - routine optometric evaluation (refraction, contact lenses, binocular vision, low vision, diagnostic procedures, paediatric optometry) - professionalism (punctuality, dress code, conduct, accountability, efficiency) - compliance (record keeping, time keeping, task completion, case reporting) - general comments
	DCT Optometrists	(11) Decentralized clinical training platform: General clinic guidelines [University 4]	Not stated	The document outlines: - the student responsibilities and conduct while on site - supervisor information on what to expect from students - supervisor instruction on how to train and supervise students - expected minimum procedures and techniques to be conducted when examining patients, as prescribed by the HPCSA (basic examination guidelines) - assessment requirements including log-sheet and marks allocation
	DCT Optometrists	(12) Decentralized clinical training platform: General clinic student assessment form [University 4]	Not stated	The document outlines requirements (clinical and cross fields) for rating the overall student performance whilst under supervision, based on the set criteria, namely: Cross fields competencies (professionalism, patient management, team work, time management, ability to work with limited resources, ability to adapt testing to case-based practice, leadership, communication with patients/peers hygiene and neatness)

**Table 2:** Quality appraisal checklist for the documents sourced

Requirement ↓	Document →	1	2	3	4	5	6	7	8	9	10	11	12
***Availability***													
Is the document readily available online/upon request?		1	1	1	1	1	1	1	1	1	1	1	1
Does the document provide full reference list?		0	1	1	0	0	0	0	0	0	0	0	0
Does the document provide a summary/goal/scope of its recommendations?		1	1	1	1	1	1	1	1	1	1	1	1
***Dates***													
Is there a completion/approval/effective date available?		0	0	1	0	1	1	0	0	0	0	0	0
Does the document provide an anticipated review date?		0	0	1	0	0	1	0	0	0	0	0	0
***Underlying Evidence***													
Does the document provide an outline of the rationale or background to the problem?		1	1	1	0	0	1	0	0	0	0	0	0
Does the document provide an outline of a strategy they used to find underlying evidence?		0	1	1	0	0	1	1	1	1	1	1	1
Does the document appraise the quality of the evidence, which underpins its recommendations?		0	1	0	0	0	1	1	1	1	1	1	1
Does the document link the hierarchy and quality of underlying evidence to each recommendation?		1	1	0	0	0	0	1	1	1	1	1	1
***Guideline Developers***													
Are the developers of the document clearly stated?		1	1	1	1	1	1	0	1	1	1	1	1
Are the qualification and/or expertise of the document developers stated, and link with the purpose of the document and its end users?		0	_0_	_0_	_0_	_0_	_0_	_0_	_0_	_0_	_0_	_0_	_0_
***Guideline Purpose and Users***													
Are the purpose and/or objectives of the document stated?		1	1	1	0	1	1	0	0	0	0	0	1
Are the target users of the document stated?		0	1	1	0	1	1	1	1	1	1	1	1
***Ease of Use***													
Is the document readable and easy to navigate?		1	1	1	1	1	1	1	1	1	1	1	1
**TOTAL SCORE**		**7**	**11**	**11**	**5**	**7**	**11**	**7**	**8**	**8**	**8**	**8**	**9**
**RATING in %**		**46.7**	**73**	**73**	**33.3**	**46.7**	**73**	**46.7**	**53.3**	**53.3**	**53.3**	**53.3**	**60**

Adapted from iCAHE guideline quality checklist template

**Table 3:** Content Analysis

No.	Code	Category	Theme
1.	Evaluation process	Process	- Required processes and procedures
2.	HPCSA requirement	Requirement	
3.	Minimum clinical practice requirements		
4.	Clinical supervision/training guidelines	Guideline	
5.	Accreditation guidelines		
6.	Reporting tools		

7.	Evaluation process	Process	- Required knowledge, skills and conduct
8.	Clinical proficiency	Skill	
9.	Communication skills		
10.	Professional conduct	Clinical conduct	- -
11.	Clinical optometry practice	Knowledge	-
12.	Clinical devices		
13.	Core competencies	Requirement	
14.	- Minimum clinical practice requirements		
15.	- Clinical supervision/training		
16.	- Optometric education		
17.	- HPCSA minimum competency requirements		
18.	- Clinical supervision/training guidelines	Guideline	
19.	Reporting tools		

## Results

To suit the intended purpose of this research the researcher adapted the iCAHE (Source 1) by increasing the questions to 15 and merged or adjusted some of the original questions (Table 2).

The comprehensive online search revealed that there are no optometry clinical training and practice documents found on the department of Health (National and Provincial) websites. Available DOH documents are mainly related to general health policies, for example, levels of care standards, primary care guide and treatment guidelines. Individual provinces are given the latitude to develop their own clinical practice standards, policies and guidelines as guided by the National Health policies. When contacted, the responsible administrative personnel informed the researchers that the facilities rely on the universities to provide student training documents. The CHE on the other hand, indicate on the website that, it does not provide training institutions with the curriculum, content, and modes of delivery or assessment criteria of a programme^3^. These are considered to be the responsibility of the awarding institution offering a programme in consultation with the communities of practice, as long as the programme meets the CHE qualification standards requirements^3^.

The researchers managed to source six relevant documents from the HPCSA and six from the four universities selected for the study. Document appraisal found (Table 2), most documents were not signed-off and did not include important information such as a reference list, rationale to the problem the document aims to address, document purpose, the development date, review date, nor qualifications of developers. When exploring the contents of the sourced documents, eighty-two meaningful units were identified and analysed, and fifteen codes emanated (Table 3). They were grouped further into six categories according to document purpose, for ease of reference. Two themes emerged, namely, the required optometry knowledge, skills and conduct, and the required optometry processes and procedures.

The reviewed HPCSA documents target different optometry audiences (Table 1) and address major, but different, aspects (Table 3). The universities documents target optometry clinical supervisors and students, including DOH optometrists (Table 1) and they describe detailed processes and procedures to follow in assisting students when examining patients, on how to perform particular tests and what to consider when recording the findings. The same documents assist the supervisors, including DOH optometrists, on what to look for when supervising students. Student assessment forms were also found amongst the reviewed documents.

## Discussion

### Documentation quality

The study results revealed that the reviewed universities documents are appropriate for optometry student clinical training and are generally aligned to each other, and with the HPCSA. As both the CHE and HPCSA allow institutional autonomy, the reviewed documents vary vastly, with each having its own strengths and limitations in terms of quality, transparency and core content. These discrepancies in documents design, content and presentation have potential for misinterpretation or non-usage by intended audience^10^. There is no uniform framework for available documents; some documents are clearly articulated, easy to follow and have a clear purpose with outcomes, interventions and recommendations, as opposed to others. Guiding documents are expected to be of good quality, be consistent, easy to use in practice, should indicate the methods used for document development, including recommendations, and provide timing of updates as well as economic and editorial independence^17^. Most of the reviewed documents were found to meet these requirements; they were accessible and coherent in relation to the core outcome competencies across universities and with the HPCSA.

**Theme 1:** Required optometry knowledge, skills and conduct

The reviewed documents revealed that HPCSA regulates the quality of optometric education in the country, defines minimum clinical practice standards and directs how practitioners should conduct themselves to remain registered to practice. In order for a student to graduate and register as an independent optometrist, there are certain clinical elements to be met, outlined as key components in the various HPCSA documents (Table 1). These include the ability to determine and perform relevant and/or appropriate methods to assess the ocular health and visual status across a full range of patients and to diagnose, and manage appropriately.

It is important for the responsible national education committees to harmonise programmes and ensure that there is alignment of policies, standards or guidelines for institutions to utilise in curriculum development and design of student clinical training programmes^17^. All the reviewed university documents (Table 1), outline clinical attributes that are aligned with the HPCSA requirements and each other. They define the standards for general and specialised optometry namely, the knowledge and skills to identify and manage refractive abnormalities/ocular pathology and other visual and binocular anomalies and treat with optical devices, vision therapy and other appropriate strategies. These elements are included in the university curricula and clinical training guidelines for undergraduate programmes and are reflected in the HPCSA documents on minimum practice requirements for all optometrists, whether in public or private clinical practice (Tables 1 and 3).

In relation to clinical training within the DOH facilities, those responsible for student clinical placements should provide both the students and supervisors with clear guidance covering detailed training expectations^19^, for successful implementation of the programme. Since this study aimed to determine how the HPCSA's PBODO and DOH standards align and guide the defined clinical training standards of the universities offering optometry programme, it is encouraging to note that the defined clinical standards of the PBODO are applicable to both students in training and to clinical trainers/supervisors involved in teaching, supervising and mentoring of students. The strong alignment of the HPCSA minimum outcome competencies for undergraduate training with minimum clinical practice requirements should serve to guide universities when designing the clinical training programme and in setting criteria for clinical supervision of students by registered practitioners. To alleviate the anecdotally reported frustrations expressed by supervisors at training sites, as mentioned in the research problem section, which needs to be probed further, it is incumbent on them to review and practice according to the minimum clinical competencies' documents of the HPCSA, irrespective of additional information or guidelines provided by the universities.

It is worth noting that, irrespective of the category of audience that the reviewed documents target, they emphasise minimum cross-field and clinical competencies, which are the core attributes required across all areas of optometry clinical education and practice. They are presented as the core set of knowledge, attributes and skills for independent, competent, ethical, safe and efficient professional practice, and are included and emphasised in each of the HPCSA documents.

Professional attributes highlighted can be summarised as having good communication and clinical skills, enabling practitioners, supervisors and students to conduct themselves professionally and ethically. They include displaying respect, confidentiality, compassion, punctuality, professional dress code, record keeping, time management, task completion, case reporting, accountability, professionalism, teamwork, roles and responsibilities, ability to improvise, leadership, cleanliness, safe environment and efficiency.

The undergraduate optometry curricula of all universities include the professional conduct and ethical framework of the HPCSA. Further, the website of the regulator contains the same documented rules and regulations, which all registered practitioners have an obligation to fulfil. It therefore, does not matter whether the target audience is an independent practitioner, student optometrist or student clinical supervisor; the expected attributes are similar (Table 3). Availability of documents relating to professional attributes and conduct (Table 2) means that both students and supervisors at DOH training sites should be aware of, and function in accordance with, the same ethical framework. To highlight the consistency of the regulator's requirements, the same attributes are required for optometrists who would like to practice as independent practitioners in SA, but qualified from universities outside SA.

**Theme 2:** Required optometry processes and procedures The review showed that there are optometry processes and procedures that need to be followed by students, optometrists and student supervisors in order to manage patients and supervise students efficiently. The strength across all documents was that the defined processes and procedures listed aim towards meeting the minimum required standards for patient care. As reflected in Table 1, there is link between the reviewed HPCSA documents for optometry professional practice and the optometry processes and procedures used by the universities, in supervision of students. The universities' documents outline the processes and procedures in detail, guided by the HPCSA documents, which only specify generic processes and procedures. The DOH as a key partner in health sciences student training, it would be expected that it has documents detailing processes and procedures, based on available HPCSA standards, for their own practice and to use in guiding student supervision at their facilities. In order to meaningfully contribute and ensure clarity of purpose, the DOH may develop specific student training guidelines and not rely solely on those documents provided by the universities. Universities, on the other hand, should include the DOH and other relevant stakeholders when developing their clinical guidelines to ensure alignment and avoid the cited frustrations of public sector optometrists.

Assessment forms reviewed differ amongst the universities although they contained detailed criteria designed according to the HPCSA outcome competencies requirements. To ensure consistency in student clinical competencies evaluations, the existing assessment documents should be shared with clinical supervisors at external training sites, in the absence of such documents. However, to maintain academic rigour and quality of assessments, the universities should determine the individual supervisor competence level and experience, and academic level of students in order to appropriately assign responsibilities and allocate students^20^. As the DOH is a critical partner in student clinical training, universities should periodically provide upskilling opportunities to clinical supervisors towards the standardization of protocols for teaching and assessment. This ultimately ensures that the high standard of care is administered to patients, who are key participants in the clinical training process.

## Strength and limitations

The opportunity to source a variety of documents used in guiding optometry student training is the study strength as it enabled empirical determination of their alignment amongst institutions, and with the HPCSA. The absence of DOH documents to guide optometry student clinical training limited comparisons, which was a study limitation. This calls for a need to conduct further studies on the topic, using other methods to obtain a deeper understanding of this important stakeholder. It is important that the documents used in optometry student training be benchmarked against those used by optometry universities in other countries as well as with those used in other health professions.

## Conclusion

It is evident from the current study that there is documentary alignment amongst the defined clinical training and supervision standards of universities offering optometric education (UL, UKZN, UJ and UFS) and HPCSA towards the delivery of quality clinical care of patients within the public facilities in South Africa. However, these documents differ from each other in terms of presentation (design and content). There is a need to have a standard format to follow in developing various documents, for each guiding document to meet the expected requirements.

There are no separate expected sets of knowledge and clinical practice standards for student optometrists and qualified optometrists involved in supervising students. The DOH optometrists, being qualified, registered and experienced should be aware of the HPCSA minimum standards and practice requirements that are available on the HPCSA website. Furthermore, they could use the regulator's and universities' documents to guide them in the development of their own sets of student training documents. It is recommended that subsequent studies be undertaken to confirm and determine the frustrations expressed by DOH supervisors.

The review has revealed a need for all stakeholders involved in student clinical training to collaborate and ultimately ensure high standards of documents for optometry clinical training are developed, in relation to the HPCSA, CHE and DOH requirements. Furthermore, the administration of quality care to patients, who are key participants in the clinical training process
